# Evidence synthesis to inform model-based cost-effectiveness evaluations of diagnostic tests: a methodological review of health technology assessments

**DOI:** 10.1186/s12874-017-0331-7

**Published:** 2017-04-14

**Authors:** Bethany Shinkins, Yaling Yang, Lucy Abel, Thomas R. Fanshawe

**Affiliations:** 1 Test Evaluation Group, Academic Unit of Health Economics, Leeds Institute of Health Sciences, University of Leeds, Worsely Building, Clarendon Way, Leeds, LS2 9LJ UK; 2 Nuffield Department of Primary Care Health Sciences, University of Oxford, Radcliffe Observatory Quarter, Woodstock Road, Oxford, OX2 6GG UK

**Keywords:** Cost-effectiveness, Diagnostic test accuracy, Health-economic model, HSROC, Meta-analysis, Threshold effects

## Abstract

**Background:**

Evaluations of diagnostic tests are challenging because of the indirect nature of their impact on patient outcomes. Model-based health economic evaluations of tests allow different types of evidence from various sources to be incorporated and enable cost-effectiveness estimates to be made beyond the duration of available study data. To parameterize a health-economic model fully, all the ways a test impacts on patient health must be quantified, including but not limited to diagnostic test accuracy.

**Methods:**

We assessed all UK NIHR HTA reports published May 2009-July 2015. Reports were included if they evaluated a diagnostic test, included a model-based health economic evaluation and included a systematic review and meta-analysis of test accuracy. From each eligible report we extracted information on the following topics: 1) what evidence aside from test accuracy was searched for and synthesised, 2) which methods were used to synthesise test accuracy evidence and how did the results inform the economic model, 3) how/whether threshold effects were explored, 4) how the potential dependency between multiple tests in a pathway was accounted for, and 5) for evaluations of tests targeted at the primary care setting, how evidence from differing healthcare settings was incorporated.

**Results:**

The bivariate or HSROC model was implemented in 20/22 reports that met all inclusion criteria. Test accuracy data for health economic modelling was obtained from meta-analyses completely in four reports, partially in fourteen reports and not at all in four reports. Only 2/7 reports that used a quantitative test gave clear threshold recommendations. All 22 reports explored the effect of uncertainty in accuracy parameters but most of those that used multiple tests did not allow for dependence between test results. 7/22 tests were potentially suitable for primary care but the majority found limited evidence on test accuracy in primary care settings.

**Conclusions:**

The uptake of appropriate meta-analysis methods for synthesising evidence on diagnostic test accuracy in UK NIHR HTAs has improved in recent years. Future research should focus on other evidence requirements for cost-effectiveness assessment, threshold effects for quantitative tests and the impact of multiple diagnostic tests.

**Electronic supplementary material:**

The online version of this article (doi:10.1186/s12874-017-0331-7) contains supplementary material, which is available to authorized users.

## Background

Just like any other intervention or medical device, diagnostic tests require rigorous evaluation before being implemented in clinical practice. Evaluations of diagnostic tests are notoriously complex, however, in part because of the indirect nature of their impact on patient outcomes.

Economic evaluations are now intrinsic to adoption decisions; many guideline bodies will not recommend a test for use in clinical practice without evidence of its cost-effectiveness [1–4]. Model-based health economic evaluations of tests have become increasingly popular as they allow many different types of evidence to be considered and incorporated, as well as enabling estimates of cost-effectiveness to be made beyond the duration of any single study. To facilitate this analysis, evidence should preferably be synthesised from all aspects of the care pathway in which the test is used.

Economic decision models typically require information about test accuracy to estimate the proportion of patients on each care pathway following a particular test outcome, and to weight the associated costs and health outcomes [1]. Test accuracy studies, however, are prone to many types of bias and formulating study designs that produce high quality evidence can be challenging [5]. Additional complexities arise when attempting to synthesise evidence from different test accuracy studies. One statistical complication is that accuracy is typically summarised using two linked, dependent outcomes: sensitivity (the accuracy of the test in patients with the target condition) and specificity (the accuracy of the test in patients without the target condition) [6]. Statistical models which account for these correlated outcomes, such as the bivariate model and the Hierarchical Summary Receiver Operating Characteristic (HSROC) model, are now advocated as best practice [5, 7–9].

In 2009, Novielli et al. conducted a systematic review of UK NIHR Health Technology Assessment (HTA) reports that had carried out economic evaluations of diagnostic tests [10]. They evaluated the methods used to synthesise evidence on test accuracy, specifically, and looked at how these results were subsequently incorporated into economic decision models. Very few of the reports implemented meta-analysis methods that accounted for the correlation between sensitivity and specificity. Since this review, significant work has been carried out to make best practice methods for the meta-analysis of diagnostic accuracy more accessible [7, 11, 12].

The authors also highlighted two potential methodological problem areas in their discussion: 1) how thresholds were dealt with in the evaluation of quantitative tests, and 2) how reviews account for the potential dependency in performance for combinations of tests [5]. The first issue relates to tests that produce numerical results which are subsequently categorised into ‘positives’ or ‘negatives’ by selecting a specific threshold. The threshold at which accuracy is reported can differ between primary studies, causing problems when pooling results in a meta-analysis. A lot of work has been carried out in recent years to develop methods to overcome this issue [13–16], but the extent to which they are being used is unclear. The second issue arises when more than one test is being evaluated within a clinical pathway and the performance of one impacts on the other (see [17] for more information).

An additional challenge we encounter frequently is the paucity of diagnostic accuracy evidence from the primary care setting. Conducting diagnostic accuracy research in primary care is challenging due to low disease prevalence, inflating both research time and costs [18]. As a consequence, much of the evidence for tests used in general practice has been acquired in secondary care settings [19]. Although it is well known that the predictive value of a test is dependent on the prevalence of the condition in question, it has only recently been acknowledged that test characteristics such as sensitivity and specificity also vary across clinical settings due to differing spectrums of disease severity [20]. These findings bring to question the transferability of evidence generated in secondary care to primary care.

The main objective here is to review current methods used to synthesise evidence to inform economic decision models in Health Technology Assessments of diagnostic tests. We update Novielli et al.’s original review, but also expand to formally assess the methodological issues outlined.

## Methods

### Inclusion criteria

We screened all 480 UK NIHR HTA reports published between May 2009 (the end date of the Novielli et al. review [10]) and July 2015 (Volume 19, Issue 52 of the NIHR HTA Journals Library).

In an initial screen, we determined whether each report evaluated an intervention or treatment; evaluated a test or multiple tests; focused on methodological development; or fell into none of these categories (for example, purely observational studies such as [21]). Of those that evaluated at least one test, we classified the primary role of the test as either screening, diagnosis, prognosis, monitoring or treatment selection based on the abstract of the HTA report. The last of these categories, which was not considered by Novielli et al. [10], was included to recognise that the purposes of some HTA reports is to use test results to guide a suitable choice of treatment in a particular patient group; see [22] for an example of a report that falls into this category. The five categories are not mutually exclusive, as some reports have multiple objectives, so for the purposes of our study, precedence was given to the ‘diagnosis’ classification if a report additionally fell into one of the other categories.

We then assessed the full texts of those that evaluated a diagnostic test to identify those that included both a model-based health economic evaluation and a systematic review. Those that carried out a systematic review were categorised further according to whether they conducted a formal meta-analysis of diagnostic test accuracy results (defined as the statistical pooling of quantitative study outcomes) as part of their systematic review, which was the final criterion for inclusion.

We additionally scrutinised two groups of reports that fell outside our original inclusion criteria. Firstly, we looked at reports of diagnostic tests that conducted a systematic review but did not carry out a meta-analysis, as we were interested in the factors that led the authors of these reports to use results summaries other than meta-analysis. Secondly, we considered prognostic studies that conducted both a health economic evaluation and a meta-analysis, in the expectation that the methodology used for diagnostic studies is often equally applicable in a prognostic setting [23].

### Data extraction

A data extraction spreadsheet was developed (a list of all of the information extracted is available in the Additional file 1).

The search criteria from each systematic review was extracted to determine whether the authors specifically looked for evidence on test-related outcomes or patient outcomes, rather than only diagnostic accuracy results, when deciding which studies to include. Test-related outcomes includes items such as test failure rate, time to test result, proportion of inconclusive test results, and interpretability of the test results. Patient outcomes refers to items that capture the downstream consequences of the test for the patient, including clinical decisions in terms of further tests, treatment and management for patients, and associated health service utilization and costs, as well as patient health outcomes such as survival and/or health related quality of life. We also recorded which reviews presented information about test-related outcomes or patient outcomes in their report, whether or not these outcomes had been included in the search criteria. Thus this evidence was classified as ‘systematically searched for’, ‘some evidence reported but not systematically searched for’, or ‘neither systematically searched for nor reported’.

For studies that reported a meta-analysis, we extracted the following key information: 1) the outcomes synthesised, 2) whether a formal quality assessment of the included studies was carried out, 3) the statistical methods used to synthesise test outcomes, and 4) whether these pooled outcomes were included in the subsequent health economic analyses. If a report contained more than one diagnostic test, it was regarded as having included the relevant information if this was reported in any of the tests considered.

For the group of studies that carried out a systematic review but did not present a meta-analysis, we reviewed the methods section of the report to ascertain if a meta-analysis had been planned and, if so, the reasons it was not carried out or not reported.

Apart from the above, we were also particularly interested in three other factors and extracted data on: 1) how threshold effects were explored/analysed in evaluations of tests that produce quantitative results, 2) how evidence from different healthcare settings from the one of interest was handled, and 3) how reviews incorporated information from multiple tests as part of a treatment pathway.

The whole screening and extraction process was carried out independently by two researchers (BS and TRF). If required, disagreements were resolved by an additional researcher (LA or YY).

## Results

### Search results

**Fig. 1:**
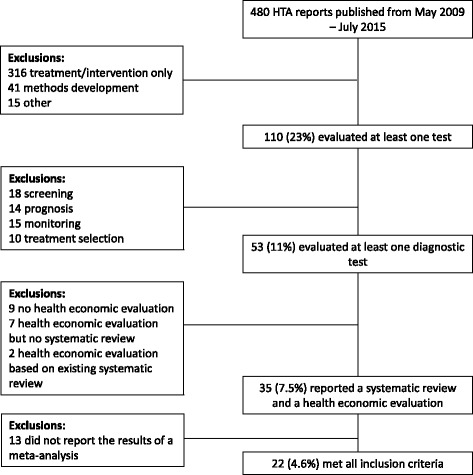
Flowchart of screening strategy

A flowchart of the search results and reasons for inclusions can be found in Fig. 1. Of the 480 UK NIHR HTA reports considered, 110 (23%) evaluated a test, and around half of these (53) were for the purpose of diagnosis. The 18 reports of diagnostic tests that were excluded because they did not include a systematic review tended to include primary research which informed the economic model parameters. A total of 35 reports [24–58] included a health economic evaluation of at least one diagnostic test and a systematic review, and 22 of these [37–58] reported the results of a meta-analysis as part of their systematic review. Additionally, there was a single report of a prognostic test, a study of foetal fibronectin testing to predict pre-term birth [59], that included both an economic evaluation, a systematic review, and a meta-analysis of the accuracy of the prognostic test.

### Evidence reviewed in addition to diagnostic accuracy

In addition to diagnostic accuracy outcomes, evidence on test-related outcomes (8/22 reports) [37, 44, 45, 47, 50, 51, 56, 57] and patient outcomes (11/22 reports) [37, 44, 45, 47, 49–51, 54, 56–58] was also explicitly searched for in the systematic reviews. Additionally, four reports presented information about test-related outcomes [40, 52, 53, 55] and four presented information about patient outcomes [39, 52, 53, 55] without these being systematically searched for, according to the search criteria. When available, evidence on test-related outcomes was summarised descriptively. Patient outcome evidence was often not available, or was available from only one or two studies, which were not always randomised trials. Of the fifteen reviews that reported patient outcome data, only three identified sufficient evidence for meta-analysis [44, 49, 53]; the remainder identified no or little evidence, thereby negating the possibility of meta-analysis.

### Meta-analysis methods and reporting

Of the 13 reports that carried out a diagnostic accuracy systematic review but did not present a meta-analysis, a meta-analysis had been planned but not carried out in all but two: one of these was an initial scoping exercise [24], and the other presented pooled summary estimates of treatment effectiveness but not the diagnostic test under consideration [36]. Of the other 11, a high level of clinical and/or statistical heterogeneity was the primary explanation most commonly given for not reporting a meta-analysis, although some reported finding insufficient or inadequate data in the primary studies [29, 30, 35] and one cited “the overwhelming positive nature of all of the results regarding the analytical validity of each of the tests” [28].

In all 22 reports meeting the inclusion criteria, the QUADAS or QUADAS2 checklist (depending on the timing of the report) was used to evaluate the quality of the diagnostic accuracy studies included in the reviews [60]. Table 1 shows the statistical methods used to synthesise the evidence on test accuracy and the pooled summary statistics presented following meta-analysis.

**Table 1:** Summary of methods and reporting used in the meta-analysis of diagnostic accuracy data

Assessment Item	No. of meta-analyses (/22)
Primary model used to pool accuracy data	
Bivariate or HSROC model	20
Independent models for sensitivity and specificity	2
Pooled accuracy statistics reported	
Sensitivity and specificity	22
Positive and negative likelihood ratio	11
Positive and negative predictive value	1
Diagnostic odds ratio	9
Area under the ROC curve	1
Exploration of Heterogeneity	
Subgroup analysis	14
Multivariable adjustment	3
Heterogeneity quantified (e.g. using I^2^ measure)	11
Presentation of Results	
Forest plot	16
Plot in ROC space/summary ROC curve	18
Investigation of Threshold Effects	
Evaluated a quantitative index test	7 (excluding 1 IPD analysis)
Existence of threshold effect clearly identified	4
Clear threshold recommendations presented	2

The bivariate model or the equivalent HSROC model (including variants that use a Bayesian framework) were implemented for the primary analysis in all but two reports [42, 57]. Many of these reports had to resort to statistical models that do not account for the correlation between sensitivity and specificity (e.g. fitting two separate random or fixed effects logistic regressions for sensitivity and specificity, setting the correlation parameter to zero in the bivariate model) for some secondary meta-analyses due to the small number of studies or convergence issues.

Forest plots depicting the between-study heterogeneity of sensitivity and specificity estimates were presented in 16 of the 22 reports, and estimates from contributing studies were displayed in ROC space (with or without an overlaid fitted summary ROC curve) in 18 reports. Other less common graphical methods used include Fagan’s nomogram for likelihood ratios [55], a plot to show the change in sensitivity and specificity according to changes in the quantitative threshold [44], and a graph of pre-test against post-test probabilities [48]. Formal quantification of heterogeneity using I^2^ or the chi-squared test was presented in 11 reports.

A wide variety of software was used to carry out the meta-analyses, Stata (in particular, the glamm and metandi functions [11]) being the most popular (used in 11/22 studies). Other software packages used include MetaDiSc, R (the now defunct diagmeta package), Review Manager, SAS (the METADAS and NLMIXED macros) and WinBUGS.

## Summary of other outcomes

### Investigation of Threshold Effects

Seven of the 22 reports evaluated at least one diagnostic test that produced fully quantitative results [38, 40, 43, 44, 52, 55, 58]. Additionally, one report used an individual participant data (IPD) meta-analysis [48] which, by its nature, allows a range of quantitative thresholds to be considered and which is discussed separately below. Two other reports used a test that was partially quantitative, in the sense that the diagnostic decision was based on both quantitative and qualitative information, but the final decision could not be expressed based simply on a numerical score or measurement [37, 42].

Accuracy was reported at differing thresholds across primary studies within each of the seven diagnostic reports that evaluated quantitative tests. Five of these reports found at least one study in their systematic review that reported diagnostic accuracy at more than one threshold. All but one of these reports [43] showed a summary ROC curve to depict how accuracy varied at different thresholds. Six attempted to quantify threshold effects – the tendency of diagnostic performance to vary according to the threshold used to define test positivity – but only two [44, 55] were able to provide unambiguous recommendations about the threshold practitioners should use. Even among these, one [55] was hindered by limited primary evidence about the performance of faecal calprotectin for the diagnosis of inflammatory bowel disease at thresholds other than 50 μg/g, and in the other [44] the authors were able to investigate threshold effects for only one (the Edinburgh Postnatal Depression Scale) of the 14 measures considered for the identification of postnatal depression in primary care.

The single relevant report that considered a prognostic score found that all primary studies in the systematic review that reported the threshold used the same standard foetal fibronectin value of above 50 ng/ml to define a positive test result.

### Individual patient data (IPD) meta-analysis

In one report [48], which looked at patient signs and symptoms as factors in the diagnosis of heart failure, the authors wanted to explore the diagnostic potential of several variables simultaneously, with the restriction that variables were required to be obtainable in a general practice setting. In many scenarios, the performance of diagnostic tests are reported on a ‘per variable’ basis, without any attempt to perform multivariable adjustment or create a combined diagnostic score. In contrast, several previous heart failure diagnostic and prognostic models had previously been developed [61], some of which found widespread use, although the large number of variables required to be collected means that not all are suitable for primary care.

To overcome this issue, the authors obtained full patient-level data from nine of the primary cohorts that they had identified in their systematic review. They then used these in an IPD meta-analysis to create a series of seven new candidate diagnostic scores using logistic regression modelling, although the presence of heterogeneity meant that data from only one of the primary studies were used to develop the model; data from the other studies were used for validation. The IPD allowed the authors not only to develop the diagnostic models, but also to test the resulting scores were adequately calibrated and to investigate the effect of changing the threshold for test positivity on diagnostic performance, with a view to creating a usable set of decision rules suitable for general practice. Results are presented in terms of pre-test and post-test probabilities of heart failure.

### Health care setting

The report of Mant et al. was one of only three [44, 45, 48] in our final sample that aimed unambiguously to assess a diagnostic test for use in primary care or as a point-of-care test, and was also unusual in that its IPD analysis specified a primary care setting as an inclusion criteria. Four other reports [40, 41, 53, 55] considered tests that were potentially usable or primary care, or which might be used across different health settings.

These reports did not restrict their search to studies conducted in primary care, and did not distinguish between setting when conducting the meta-analysis. Indeed, for most of these reports, few or no studies based in a primary care setting were found and so the authors were unable to assess whether test accuracy was the same in primary and secondary care. For example, the faecal calprotectin report of Waugh et al. [55] specifically recommends studies in primary care populations as a future research need. Similarly, Drobniewski et al. [41] discuss the need for further research in targeted patient populations for the use of molecular tests for antibiotic resistance in tuberculosis as point-of-care tests; the majority of primary studies included in that report were conducted in a laboratory environment.

### Health economic modelling

In extracting information about the types of health economic decision analytic models implemented, we found that the methodology was often described using differing terminology and reported in varying level of detail. The two most common classes of models adopted were decision trees and Markov models, although the precise form of the model varied, and other model formulations guided by the healthcare context (e.g. [43, 47, 55]) were also used.

The extent to which the results of the test accuracy meta-analyses informed the model parameters in the cost-effectiveness analyses was evaluated. Four of the test accuracy meta-analyses, including the one IPD meta-analysis [48], provided all of the accuracy data required for the cost-effectiveness modelling [38, 44, 48, 58]. In fourteen reports, some of the accuracy parameters had to be informed by single studies, expert opinion, or assumptions [39–41, 43, 45–47, 50–53, 55–57], including one in which the results of the meta-analysis had to be adjusted to account for the fact that the reference standard used was not 100% accurate [46]. In four reports, the results of the diagnostic accuracy meta-analysis were not used to inform the cost-effectiveness analyses. Instead, accuracy model parameters were either extracted from single studies [37, 54] or elicited from clinicians [49], or based on a combination of these [42].

In all 22 of the reports, some attempt was made to explore the effect of uncertainty in the accuracy estimates, typically via one-way (univariate), extreme case or, more commonly, probabilistic sensitivity analysis.

Of the seven reports that evaluated the cost-effectiveness of a fully quantitative test, the majority (6/7) explored how differing the threshold impacted on the cost-effectiveness of the test. This was, however, restricted to the few thresholds at which sufficient data was available for meta-analysis or a sub-group analysis. In contrast, threshold was included as a full parameter in the cost-effectiveness model where IPD was available and ‘optimised’ against willingness to pay [48].

### Multiple tests within the diagnostic pathway

The majority of reports (16/22) considered the effect of a combination of tests within the diagnostic pathway into their decision model. This includes both scenarios in which a new test is being added to an existing one, possibly at a different point in the pathway and those in which multiple tests are evaluated concurrently. Most of these reports did not discuss the issue of possible dependence between multiple tests being evaluated on the same individual, or made an explicit assumption that the tests were being regarded as independent, calculating required joint probabilities via Bayes rule (e.g. [53]). Two reports tested the assumption of independence in a sensitivity analysis [46, 52].

## Discussion

This review assesses current methods used to synthesise evidence to inform economic decision models in Health Technology Assessments of diagnostic tests.

In six years of UK NIHR HTA reports published since 2009, there has been a notable improvement in the quality of meta-analytic methods used by authors. During the period 1997-May 2009, only two of fourteen reports used the bivariate or the HSROC model for meta-analysing diagnostic accuracy data across studies [10]. In the subsequent period, to July 2015, this figure rose to 20 out of 22. Our conclusion is that during the last six years, these methods are now routinely accepted as standard practice within UK NIHR HTAs. Priority areas for methodological improvement within future UK NIHR HTAs of diagnostic tests are outlined below.

### Priority area 1: Evidence requirements for cost-effectiveness evaluations of tests

Most reports focused their systematic reviews on test accuracy. Many did look for other outcomes at the same time, but not all. It was difficult to formally assess whether each systematic review had looked for evidence on all the necessary outcomes to inform the cost-effectiveness model as there is poor agreement on what evidence is required to inform adoption decisions for tests, and the evidence required is likely to depend heavily on the context. One systematic review identified 19 different ‘phased evaluation schemes’ for medical tests [62].

Nevertheless, the distinct lack of evidence identified on the impact of a test on patient outcomes was notable. Randomised controlled trials in this setting are often impractical due to long follow-up periods required to capture downstream patient outcomes, large sample sizes and the speed at which technology is advancing in this area (see [63] for further discussion on this topic). Often the technology has developed or changed by the time the trial is complete [64]. To overcome this issue, some studies have used a linked evidence approach (e.g. [65]) and carried out two separate reviews – one looking at outcomes related to the test and another focusing on the impact of therapeutic changes on morbidity, mortality and adverse effects.

There currently lacks a clear framework that describes the evidence required for cost-effectiveness evaluations of tests. For the time being, we would recommend that authors use this checklist [66] to identify outcomes potentially relevant to their research question and tailor their systematic literature searches accordingly. The same rigour implemented for the systematic searches for test accuracy evidence should also be applied for other outcomes, if the objective is to evaluate the diagnostic pathway holistically.

### Priority area 2: The evaluation of threshold effects in quantitative tests

Ideally, thresholds should be selected that balance the repercussions of a false negative and false positive result in terms of patient outcomes and costs. The accuracy of a diagnostic test is typically evaluated by calculating paired summary statistics from a 2 × 2 classification table (e.g. sensitivity and specificity). Even for quantitative tests, clinical application often involves the selection of a single threshold to guide treatment decisions, and therefore accuracy needs to be summarised at this threshold. Perhaps for this reason, primary diagnostic accuracy often report accuracy measures at just one threshold, which can limit the opportunity to pool accuracy at all thresholds and make comparisons between them in a meta-analysis.

Of the seven (non-IPD) reports that evaluated quantitative tests, the authors were able to make clear optimal threshold recommendations in only two. In the one report that carried out an IPD meta-analysis [48], diagnostic accuracy was pooled across the whole test scale, allowing it to be incorporated as a parameter that could be optimised when fitting the cost-effectiveness model. The authors were thus able to identify and recommend the threshold which provided optimal clinical- and cost-effectiveness. The issue of threshold selection is likely to be of particular importance when assessing whether test accuracy is transferrable between different settings, such as from secondary to primary care, as health settings may differ in their patient populations and in the level of accuracy that is required for the test to be adopted.

Statistical methods have been proposed to overcome this issue [13–16], but they are generally only possible to implement if accuracy is reported at multiple thresholds within a single study, or at a broad range of thresholds across different studies. We recommend where possible that 2 × 2 data is obtained across all thresholds from each study to allow accuracy to be summarised across the whole test scale and thus available as a parameter for subsequent cost-effectiveness analyses. Alternatively, researchers might consider making entire data files available for future evidence synthesis via IPD analysis, a practice which is already encouraged or required by some journals.

### Priority area 3: The evaluation of test combinations within the diagnostic pathway

Many treatment pathways rely on results from multiple diagnostic tests, either performed in parallel or in sequence. Our results show that the majority of UK NIHR HTA reports that consider test combinations treat them as independent. The few that do consider the dependence caused by performing two or more tests on the same individual are often restricted by a lack of evidence about the extent of the dependence from primary studies.

We suggest two possible strategies for dealing with this issue in future diagnostic treatment evaluations. The first, and simplest, is to conduct sensitivity analysis assuming different quantitative estimates of within-individual correlation between test results [46, 52]. The second is to conduct primary studies, including randomised trials, which consider the entire treatment pathway as the intervention, rather than only a single component. This approach, although considered by some of the reports in our review (e.g. [50]), is likely to be costly and may be difficult to evaluate if the patient pathway is complex. At a minimum, authors of primary studies should consider reporting information about between-test correlation or making IPD data available. Additionally, although some methodological work has been conducted in this area [67], further research would be valuable.

### Limitations

By including a systematic review as one of the requisites for inclusion in this review, 7 reports that evaluated at least one diagnostic test were excluded from this review. Of these, 6 were based on primary studies conducted as part of the UK NIHR HTA grant (5 randomised trials [68–72] and 1 diagnostic accuracy study [73]) and a further report based their evaluation on a sub-study of an existing trial [74].

## Conclusions

The results of this review demonstrate that, within UK NIHR HTAs, diagnostic accuracy meta-analyses are now routinely conducted using statistically appropriate methods that account for the nuances and challenges unique to diagnostic accuracy research.

Despite this commendable progress, there is still room for improvement in the methodology applied within HTAs. There is generally a gap in understanding the evidence requirements to inform cost-effectiveness analyses of diagnostic tests. More specifically, the evaluation of quantitative tests remains a challenge due to incomplete reporting of accuracy across thresholds. Greater efforts are also required to ensure that potential dependencies in test performance are accounted for when tests are used sequentially within a diagnostic pathway.

## Additional file

Additional file 1: Inclusion criteria and data extraction details. Initial screening criteria and the data extracted from reports meeting the inclusion criteria. (DOCX 16 kb)

## Abbreviations

HSROC: Hierarchical summary receiver operating characteristic
HTA: Heath technology assessment
IPD: Individual patient data
ROC: Receiver operating characteristic.
