# Supplementary material for: Evidence synthesis to inform model-based cost-effectiveness evaluations of diagnostic tests: a methodological review of health technology assessments
Source: BMC Med Res Methodol. 2017 Apr 14;17:56. doi: 10.1186/s12874-017-0331-7 (PMC5391551; doi:10.1186/s12874-017-0331-7)
Supplement: Additional file 1: — Inclusion criteria and data extraction details. Initial screening criteria and the data extracted from reports meeting the inclusion criteria. (DOCX 16 kb) [file 12874_2017_331_MOESM1_ESM.docx]

**Inclusion criteria and data extraction details**

Initial screening criteria:

- Assessment Type – Treatment alone/Testing/Methods/Other
- Testing purpose – Diagnosis/Monitoring/Screening/Prognosis/Treatment selection
- Was a cost-effectiveness analysis carried out?
- Did the grant include primary research?
- Was a systematic review carried out?
- Was a diagnostic accuracy meta-analysis carried out?
- Reasons for not doing DA meta-analysis

Data Extracted from reports meeting the inclusion criteria:

- Evidence searched for in systematic review
- Evidence identified in systematic review
- Was a quality assessment of included studies carried out? Which checklist/guidance used?
- Statistical methods used to pool diagnostic accuracy data
- Which accuracy outcomes were pooled?
- Graphical methods used to report results of diagnostic accuracy meta-analysis
- Was between-study heterogeneity explored/formally quantified? Using which methods?
- Which software was used for meta-analysis?
- Did the test under evaluation produce quantitative results?
- How were threshold effects explored/handled?
- Which evaluated tests targeted for the primary care setting?
- Was evidence collected from healthcare settings that differed to the one of interest? If so, how was this handled in the meta-analysis?
- Type of cost-effectiveness model implemented
- Were the results of the diagnostic accuracy meta-analysis used to inform parameters of the cost-effectiveness model? If not, what other evidence was used instead?
- Was the impact of uncertainty in accuracy parameters explored? Using which methods?
- How were threshold effects handled in the cost-effectiveness analysis for quantitative tests?
- Were combinations of tests considered in the cost-effectiveness analysis? If so, how was the potential correlation between them handled?
